# Effect of Essential Oil Components on the Activity of Steroidogenic Cytochrome P450

**DOI:** 10.3390/biom14020203

**Published:** 2024-02-08

**Authors:** Katyayani Sharma, Angelo Lanzilotto, Jibira Yakubu, Søren Therkelsen, Clarissa Daniela Vöegel, Therina Du Toit, Flemming Steen Jørgensen, Amit V. Pandey

**Affiliations:** 1 Division of Endocrinology, Diabetology and Metabolism, Department of Pediatrics, University Children’s Hospital, Inselspital, University of Bern, 3010 Bern, Switzerland; katyayani.sharma@unibe.ch (K.S.); angelolanzilotto@gmail.com (A.L.); jibira.yakubu@unibe.ch (J.Y.); fsx728@alumni.ku.dk (S.T.); therina.dutoit@unibe.ch (T.D.T.); 2 Translational Hormone Research Program, Department of Biomedical Research, University of Bern, 3010 Bern, Switzerland; clarissa.voegel@insel.ch; 3 Graduate School for Cellular and Biomedical Sciences, University of Bern, 3012 Bern, Switzerland; 4 Department of Drug Design and Pharmacology, University of Copenhagen, 2100 Copenhagen, Denmark; fsj@sund.ku.dk; 5 Department of Nephrology and Hypertension, University Hospital Inselspital, University of Bern, 3010 Bern, Switzerland

**Keywords:** EDCs, prostate cancer, CYP17A1, steroidogenesis, DHEA, anti-cancer drugs

## Abstract

Endocrine-disrupting chemicals (EDCs) may impact the development of prostate cancer (PCa) by altering the steroid metabolism. Although their exact mechanism of action in controlling tumor growth is not known, EDCs may inhibit steroidogenic enzymes such as CYP17A1 or CYP19A1 which are involved in the production of androgens or estrogens. High levels of circulating androgens are linked to PCa in men and Polycystic Ovary Syndrome (PCOS) in women. Essential oils or their metabolites, like lavender oil and tea tree oil, have been reported to act as potential EDCs and contribute towards sex steroid imbalance in cases of prepubertal gynecomastia in boys and premature thelarche in girls due to the exposure to lavender-based fragrances. We screened a range of EO components to determine their effects on CYP17A1 and CYP19A1. Computational docking was performed to predict the binding of essential oils with CYP17A1 and CYP19A1. Functional assays were performed using the radiolabeled substrates or Liquid Chromatography–High-Resolution Mass Spectrometry and cell viability assays were carried out in LNCaP cells. Many of the tested compounds bind close to the active site of CYP17A1, and (+)-Cedrol had the best binding with CYP17A1 and CYP19A1. Eucalyptol, Dihydro-β-Ionone, and (−)-α-pinene showed 20% to 40% inhibition of dehydroepiandrosterone production; and some compounds also effected CYP19A1. Extensive use of these essential oils in various beauty and hygiene products is common, but only limited knowledge about their potential detrimental side effects exists. Our results suggest that prolonged exposure to some of these essential oils may result in steroid imbalances. On the other hand, due to their effect on lowering androgen output and ability to bind at the active site of steroidogenic cytochrome P450s, these compounds may provide design ideas for novel compounds against hyperandrogenic disorders such as PCa and PCOS.

## 1. Introduction

**Figure 1:**
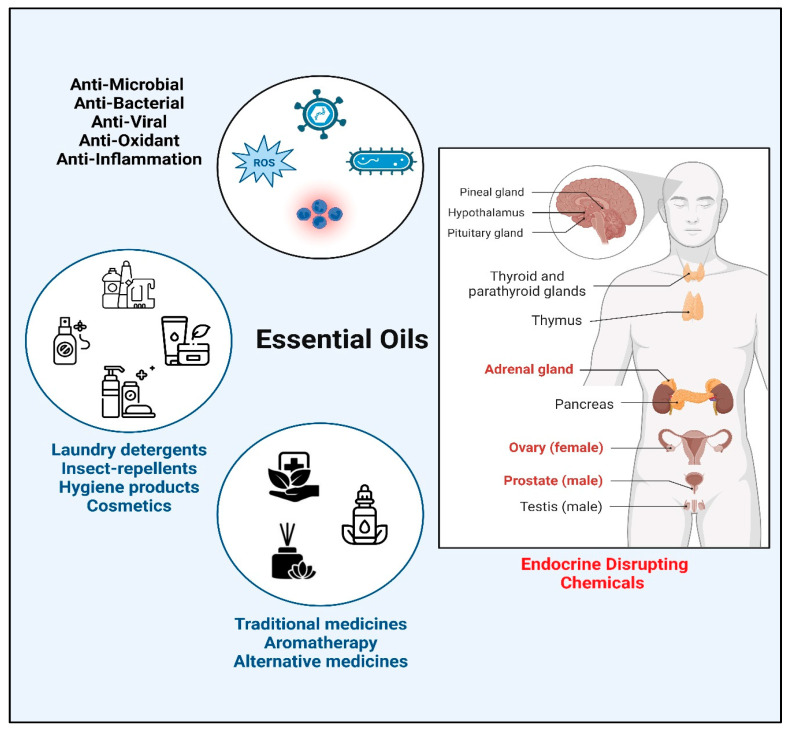
An overview of conventional usage and biological activities of essential oil components. Created by https://www.Biorender.com.

Essential oils are a complex mixture of volatile compounds extracted from aromatic plant tissues with a characteristic “essence” or smell [1]. Pure extracts of essential oils are obtained through different methods such as steam distillation, solvent extraction, and hydro distillation [2]. The chemical composition of essential oils can vary depending on the origin and species of the plant, climate, and extraction method [3]. Two major constituents of essential oils are terpenes and terpenoids [4]; some examples of terpenes found in essential oils are cineol (Eucalyptol), linalool, pinene, limonene, thujene, bisabolene, caryophyllene, p-cymene, camphor, neral, menthol, and geraniol, while aromatic compounds consist of carvacrol, thymol, cinnamaldehyde, eugenol, and estragole [5]. Owing to their fragrance, essential oils are extensively used in many cosmetic and hygiene products [6,7]. Due to their anti-microbial, antibiotic, antiviral, antioxidant, and anti-inflammatory properties, essential oils have been part of traditional therapies and herbal medicines for a long time [8,9,10] (Figure 1). Being “natural” in origin, essential oils are often considered as safer substitutes of chemical drugs that may have adverse side effects [11]. However, in addition to their therapeutic role, essential oils might function as potential endocrine-disrupting chemicals (EDCs).

EDCs are chemical substances that can alter endocrine function by interfering with steroid metabolism, resulting in hormonal imbalance in the body. Abnormal levels of steroids, especially sex steroids, can cause detrimental effects on sexual development and possess an increased risk of infertility [12], though the exact mechanism of action of EDCs is not fully known. Clinical case reports have linked prepubertal gynecomastia in boys and premature thelarche in girls to prolonged use of lavender and tea tree oil-based fragrant products which resolved upon cessation of the products. Moreover, studies in human breast cancer cell lines have shown estrogenic and anti-androgenic activity of some essential oils [8,13]. 

**Figure 2:**
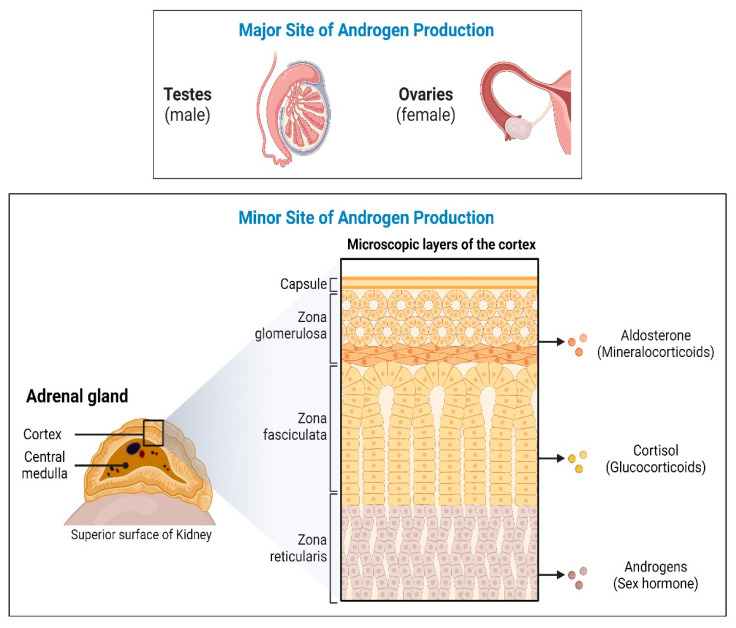
Androgen production in humans. In humans, androgens are mainly produced in the male testis and female ovaries, while androgen precursors are mainly produced in adrenal glands. Sexual traits and development as well as influence on female sexual behavior may be regulated by androgens. Androgen precursors, dehydroepiandrosterone (DHEA) and its sulfate DHEA(S), are produced in the zona reticularis of the adrenal cortex and are then converted into active androgens by the action of a series of steroid-metabolizing enzymes. Created by https://www.Biorender.com.

In humans, androgens are primarily produced in the male testis, female ovaries, and adrenal glands (Figure 2). Androgens control male sexual traits and development, as well as influence female sexual behavior. The zona reticularis of the adrenal cortex produces dehydroepiandrosterone (DHEA) and its sulfate DHEA(S). DHEA acts as a precursor to produce androgens (testosterone and androstenedione). The first, and rate-limiting, step in the biosynthesis of all steroid hormones is the cleavage of the cholesterol side chain by the mitochondrial P450 enzyme CYP11A1 to convert cholesterol into pregnenolone (Preg/P5) [14]. Further, in multi-enzymatic steps, Preg is converted into mineralocorticoids, glucocorticoids, and androgens. 

CYP17A1 [15,16] is an essential enzyme that plays a vital role in adrenal androgen production [16,17]. CYP17A1 catalyzes both the 17α-hydroxylase and 17,20 lyase reactions [18]. The 17,20 lyase activity of CYP17A1 is supported by at least three factors; the amount of P450 Oxidoreductase (POR) for electron transfer [19,20], presence of cytochrome b_5_ (CYB5A) [21,22], and the phosphorylation of the CYP17A1 protein [22,23,24,25,26]. Understanding the mechanisms of 17,20 lyase activity is important for the understanding of hyperandrogenic disorders such as PCa and PCOS [27,28].

**Figure 3:**
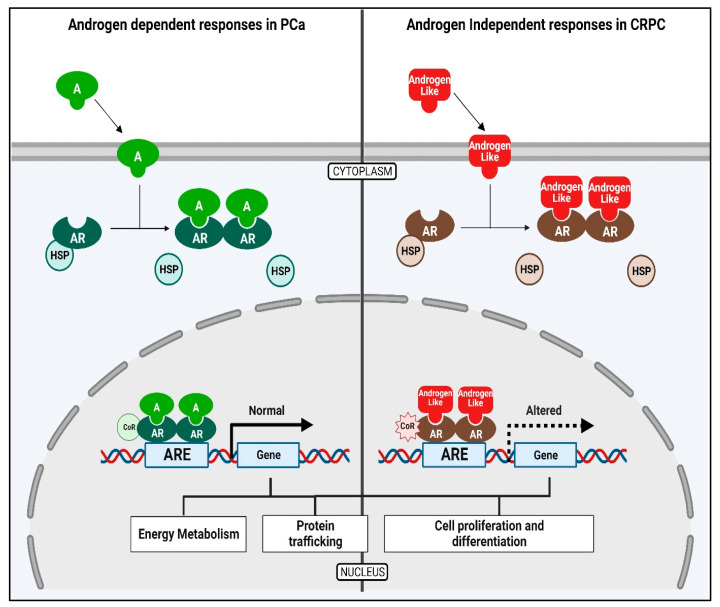
PCa cells are driven by the binding androgens to the androgen receptor (AR). The AR then acts as a transcriptional activator for the expression of genes that are responsible for the growth and progression of the PCa cells. In addition, chemicals resembling androgen-like structures may also bind to and activate androgen receptors and create signaling events similar to the effects of androgens. Created by https://www.Biorender.com.

Many chemicals with androgen-like structures may bind to and activate androgen receptors and act like EDCs (Figure 3) Epidemiological studies suggest that EDCs may function as hormone mimics and bind to nuclear receptors like the AR to elicit altered expression of genes involved in the development and progression of PCa [29] (Figure 3). The AR acts as a transcriptional activator for the expression of genes responsible for the growth and survival of the tumor [30]. Previous studies suggest that the possible role of essential oils could be either the direct inhibition/activation of steroidogenic enzymes or by the regulation of gene expression of steroid-metabolizing enzymes resulting in abnormal androgen levels in the body. High levels of circulating androgens are linked to both PCa and Polycystic Ovary Syndrome (PCOS) [31]. 

Considering the potential role of essential oils as EDCs, we explored the potential of some common essential oil components on human steroid-metabolizing enzymes, CYP17A1 and CYP19A1, for the potential effects on androgen and estrogen production in humans and as potential structural leads for the design of novel chemicals targeting these enzymes in hormone-dependent cancers. We docked multiple terpene compounds that are naturally present in essential oils into the structures of the steroid-metabolizing enzymes CYP17A1 and CYP19A1 in order to evaluate their binding modes and sites to identify possible endocrine-disrupting properties of these compounds. 

## 2. Materials and Methods

### 2.1. Terpenes

The terpenes used in the experiments cannot be called essential oils as they do not exist as mixtures of compounds but are commercially available as single chemical entities (sometimes multiple isomers coexist because the separation process can bring purity only to a certain point). The terpenes we sourced are of mixed natural/synthetic origin depending on which provided higher purity. Their isolation usually consisted of an essential oil first collected through steam distillation or alcoholic extraction of the dry plant/flower mass and then fractional distillation to collect the main components.

All terpenes were sourced from Sigma-Aldrich (St. Louis, MO, USA), unless otherwise indicated, and the individual product purity was between 90 and 99%. The product codes were: (−)-α-Pinene (305715, (purity 99%), (+)-α-Pinene (268070, purity 99%), α-Ionone (I12409, purity 90%), Benzaldehyde (B1334, purity 99%), p-Anisaldehyde (A88107, purity 98%), 1,4-Cineole (W365820, purity 95%), Isoamyl acetate (W205532, purity 97%), Octyl acetate (W280607, purity 98%), Benzyl acetate (B15805, purity 99%), Propyl acetate (133108, purity 99%), β-Pinene (402753, purity 99%), Bisabolene (Alfa Aesar, A18724, mixture of isomers), (−)-α-Bisabolol (14462, purity 93%), 3-Carene (115576, purity 90%), (S)-(+) Carvone (435759, purity 96%), (+)-Cedrol (22135, purity 99%, sum of enantiomers), Cinnamyl alcohol (108197, purity 98%), p-Cymene (C121452, purity 99%), Dihydrocarvone (218286, purity 98%, mixture of isomers), Dihydro-β-ionone (W362603, purity 90%), Eucalyptol (C80601, purity 99%), Farnesol (W247804, purity 95%, mixture of isomers), Geraniol (163333, purity 98%), Methyl anthranilate (W268208, purity 98%), (R)-(+)-Limonene (183164, purity 98%), (+−)-Citronellal (27470, purity 95%), (R)-(−) Carvone (124931, purity 98%), (1R)-(−) Myrtenal (218243, purity 98%), Nerol (268909, purity 97%), Ocimene (W353977, purity 90%, mixture of isomers), (S)-(−)-Limonene (218367, purity 96%), Carvacrol (282197, purity 98%), (+)-Sabinene (W530597, purity 75%), (S)-(−) Perillyl alcohol (218391, purity 96%), Estragole (A29208, purity 98%), (−)-α-Terpineol (W304522, purity 96%), Terpinolene (W304603, purity 95%), Thymol (T0501, purity 98%), Vanillin (V1104, purity 99%), Methyl salicylate (M6752, purity 99%), α-Terpinyl acetate (W304799, purity 95%), α-Phelladrene (W285611, purity 85%), γ-Terpinene (223190, purity 97%). 

### 2.2. Molecular Docking Analysis

In a first attempt, 82 terpene compounds were docked into CYP17A1 and CYP19A1 using AutoDock VINA (The Scripps Research Institute, La Jolla, CA, USA) [32,33]. The dataset for docking was larger than the available compound library used for testing and included structurally similar compounds and racemic duplicates to expand the virtual screening. Three reference compounds were used as controls. Ligands co-crystallized with the PDB structures [34] in PDB IDs 4NKZ [35] and 3S79 [36,37,38] (CYP17A1 and CYP19A1, respectively) were removed, and the remaining protein structures were used for docking. Three-dimensional structures of the ligands were extracted from PubChem and prepared for docking using the LigPrep [39] function within Maestro [Schrödinger Release 2022-3: Maestro, Schrödinger, LLC, New York, NY, USA, 2021], removing possible salts and ensuring generation of possible ionization and tautomeric states at pH = 7 ± 1 using the Epik [40] setting. Prior to docking, the compounds were subjected to a short energy minimization. As reference compounds, abiraterone and 17α-hydroxypregnenolone were docked into CYP17A1 and androstenedione was docked into CYP19A1, and the results were compared with known structures of CYP17A1 [35,41,42] and CYP19A1 [36,37,38,43]. This yielded a global docking simulation including the whole protein structure. For each ligand, 25 docking runs were performed. The results were subjected to a cluster analysis with each cluster differing by at least 5 Å heavy-atom RMSD, representing different possible sites and modes of binding. 

**Figure 4:**
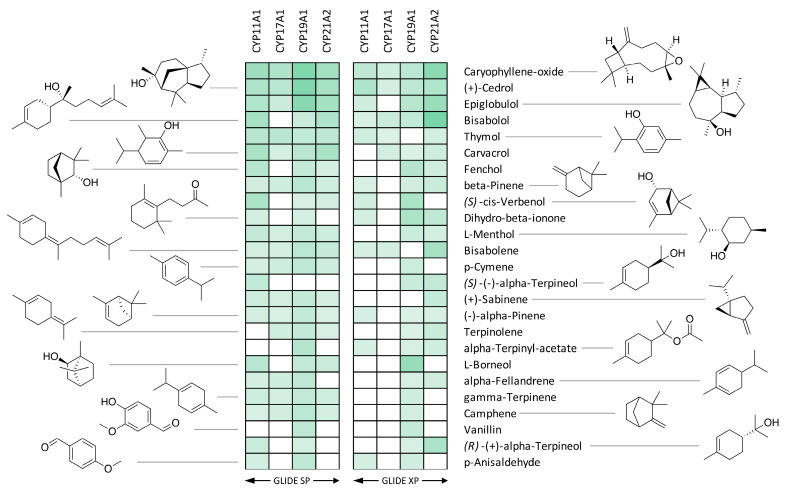
Heat maps of binding poses obtained after docking of terpenes from essential oils into steroid-metabolizing CYP enzymes. Here, we show 25 of the best binding poses. A full list is available in the supplementary materials. Binding energies vary from −8.2 kcal/mol for the best binding poses (corresponding to dark green boxes) to −3.3 kcal/mol for the poorest binding poses (white boxes).

In a further refinement of this process, the compounds were docked into not only CYP17A and CYP19A1 but also to CYP11A1 and CYP21A2 with GLIDE [v 5.8, Schrödinger, LLC, New York, NY, USA, 2021] using both the SP and XP scoring functions [44,45]. Subsequently, the best scoring poses for each compound for each enzyme and for each scoring function were extracted and analyzed and heat maps of the top 25 best scoring compounds were produced (Figure 4). A full analysis of scores is provided in the supplementary materials.

### 2.3. Chemicals

Trilostane was obtained from the extraction of commercially available tablets as Modrenal^®^ (Bioenvision, New York, NY, USA). Abiraterone acetate was purchased from MedChemExpress^®^, Lucerna Chem AG (Lucerne, Switzerland). The commercially available drug Anastrazole was purchased from AstraZeneca (Cambridge, UK). The radiolabeled substrates, Progesterone [4-^14^C] (Specific activity 55 mCi/mmol; concentration. 0.1 mCi/mL); 17α-Hydroxypregnenolone [21-^3^H] (Specific activity 15 Ci/mmol; concentration. 1 mCi/mL); and Androstenedione [1β-^3^H(N)] (Specific activity 24 Ci/mmol; concentration. 1 mCi/mL) were obtained from American Radiolabeled Chemicals Inc. (St. Louis, MO, USA). The non-radiolabeled standard substrates, Pregnenolone; Progesterone; 17α-Hydroxypregnenolone; 3-(4,5-Dimethyl-2-thiazolyl)-2,5-diphenyl-2H-tetrazolium bromide (MTT); Resazurin sodium salt; Dimethyl sulfoxide (DMSO); and Dextran were purchased from Sigma-Aldrich^®^ (St. Louis, MO, USA). NADPH tetrasodium salt and organic solvents such as isooctane, ethyl acetate, and chloroform/trichloromethane were acquired from Carl Roth^®^ GmbH + Co. KG (Karlsruhe, Germany). Activated charcoal was obtained from Merck AG (Darmstadt, Germany).

### 2.4. Cell Line and Culture

The current standard model system to study molecular and biochemical mechanisms of steroidogenesis is the NCI H295R cell line [46,47]. These cells express genes from all three zones of the adrenal cortex, providing an excellent system that closely reflects human adrenal physiology [14]. The human adrenocortical carcinoma cell line NCI H295R was obtained from the American Type Culture Collection (ATCC^®^ CRL2128™), Manassas, VA [46,47]. Cells between passages 12 and 24 were cultivated in DMEM/Ham’s F-12 medium (1:1 Mix) supplemented with L-glutamine and 15 mM HEPES (Gibco™, Thermo Fisher Scientific, Waltham, MA, USA), along with 5% Nu-Serum I; 0.1% insulin, transferrin, selenium in the form of ITS Premix (Corning™, Manassas, VA, USA) and 1% Penicillin-Streptomycin (Gibco™, Thermo Fisher Scientific, Waltham, MA, USA) at 37 °C in a humid atmosphere with a constant supply of 5% carbon dioxide to maintain the physiological pH. The human PCa cell line, derived from metastatic site, left supraclavicular lymph node, LNCaP clone FGC (ATCC^®^ CRL1740™), was cultured in RPMI-1640 Medium containing 2 mM L-glutamine with 10 mM HEPES, 1 mM Sodium pyruvate, 10% Fetal Bovine Serum, and 1% Penicillin-Streptomycin as supplements (Gibco™, Thermo Fisher Scientific, Waltham, MA, USA). For experiments, cells with passage numbers 12–30 were used as previously described [48].

### 2.5. Cell Viability Assays

To determine the effect of test compounds on the cellular activity of human adrenal NCI H295R cells, MTT-based cell viability assay was performed [49,50]. In a 96-well plate, about 30,000 cells per well were seeded with complete medium. After 24 h, the medium was replaced with fresh medium and 10 µM of test compounds was added. DMSO (less than 1% *v*/*v*) was used as vehicle control. Abiraterone (10 µM) was used as a positive control [51,52]. A total of 0.5 mg/mL MTT reagent was added to the culture medium for another 4 h. After the incubation, the medium was entirely replaced with DMSO to dissolve the formazan crystals. After 20 min, absorbance was measured at 570 nm (SpectraMax M2, Bucher Biotec, Basel, Switzerland). Percent viability was calculated with respect to the mean value of control samples. 

For PCa LNCaP cells, Resazurin-based Alamar blue assay was performed to evaluate the cell toxicity [49,50]. Cells seeded at a density of 10,000 cells per well were treated with test compounds and the controls for 24 and 48 h. After incubation, 0.05 mg/mL Resazurin in phosphate buffer was added. Cells were incubated for another 4 h in the dark at 37 °C. Fluorescence was measured at an excitation wavelength of 550 nm and an emission wavelength of 590 nm. Percent viability was calculated with respect to the mean value of control samples (DMSO).

### 2.6. CYP17A1 Enzyme Assays

The CYP17A1 enzyme assays were carried out according to well-established protocols [52,53] in our laboratory. The NCI H295R cells were seeded overnight in a 12-well plate at a cell density of 0.5 × 10^6^ cells per well. The next day, 10 µM of test compounds was added to respective wells containing fresh medium and incubated for 4 h. Abiraterone and DMSO were used as reference and control, respectively. To determine CYP17A1 hydroxylase activity, cells were treated with the [^14^C]-Progesterone at a concentration of 10,000 cpm/1 µM per well [22,23,24,54]. Trilostane was added prior to the addition of test compounds and the substrate to block 3β-hydroxysteroid dehydrogenase activity [55]. Radiolabeled steroids were extracted from the media with help of Ethyl acetate and Isooctane (1:1 *v*/*v*) and separated through Thin Layer Chromatography (TLC) on a Silica gel-coated aluminum plate (Supelco^®^ Analytics, Sigma-Aldrich Chemie GmbH, Taufkirchen, Germany) [56]. TLC spots were exposed to a phosphor screen and detected by autoradiography using Typhoon™ FLA-7000 PhosphorImager (GE Healthcare, Uppsala, Sweden). Radioactivity was quantified using ImageQuant™ TL analysis software (GE Healthcare Europe GmbH, Freiburg, Germany). Enzyme activity was calculated as a percentage of radioactivity incorporated into the product with respect to the total radioactivity.

Using similar treatment conditions, [21-^3^H]-17α-hydroxypregnenolone (50,000 cpm/1 uM per well) was used as a substrate to analyze CYP17A1 Lyase activity. NCI H295R cells were treated with test compounds for 24 h before the addition of the substrate and trilostane. Tritiated water release assay was performed [57] by measuring the conversion of 17OH-Preg into DHEA. Steroids in the media were precipitated using 5% activated charcoal/0.5% dextran solution. The enzyme activity was estimated with reference to the water-soluble tritiated by-product formed in an equimolar ratio with the corresponding DHEA. The radioactivity in the aqueous phase was measured by Liquid Scintillation counting (MicroBeta2^®^ Plate Counter, PerkinElmer Inc., Waltham, MA, USA). The percent inhibition was calculated with respect to the control [58].

### 2.7. Steroid Profiling

For steroid analysis, NCI H295R cells were treated in a similar way except that 1 µM of the unlabeled substrate, pregnenolone, was used instead of radiolabeled substrates for 4 h. Steroids were measured by a Liquid Chromatography–High-Resolution Mass Spectrometry (LC-HRMS) method, as previously described and validated [59]. Briefly, steroids were extracted from 500 µL cell media aliquots, plus 38 µL of a mixture of internal standards (at 3.8 nM each), using solid-phase extraction with an OasisPrime HLB 96-well plate. Samples were resuspended in 100 µL 33% methanol and 20 µL was injected into the LC-HRMS instrument (Vanquish UHPLC coupled to a Q Exactive Orbitrap Plus, from Thermo Fisher Scientific, Waltham, MA, USA) using an Acquity UPLC HSS T3 column (from Waters, Milford, MA, USA). Data from the mass spectrometer were processed using TraceFinder 4.0 (from Thermo Fisher, Waltham, MA, USA). The lower limit of quantification (LOQ) for pregnenolone was 0.77 nmol/L, for DHEA it was 0.85 nmol/L, for DHEA-S it was 6.25 nmol/L, and for 17OH-pregnenolone (quantified relative to the calibration of progesterone using a calculated response factor) it was 20 nmol/L.

### 2.8. CYP19A1 Assay

Estrogens are synthesized from androgens through the action of the enzyme CYP19A1 (Aromatase) [60]. We used 40 µg of microsomal proteins from placental JEG-3 (Human Choriocarcinoma; ATCC^®^ HTB36^™^) cells in 100 mM potassium phosphate buffer (pH 7.4) containing 100 mM NaCl in a reaction mixture of 200 µL to carry out Aromatase enzyme activity assay. For determining the impact on aromatase activity, 10 µM of test compounds, DMSO as a negative control, and anastrozole (a known CYP19A1 inhibitor) as positive control were added to the reaction mixture. Tritium-labelled androstenedione (~30,000 cpm/µL/50 nM) was used as the substrate to monitor the enzyme activity. The chemical reaction was initiated by the addition of reduced Nicotinamide adenine dinucleotide phosphate (NADPH), followed by incubation at 37 °C with constant shaking for 1 h. The reaction was stopped by the addition of charcoal/dextran solution. Enzyme activity was measured using a tritiated water release assay, as described earlier [53,61]. 

### 2.9. Statistical Analysis

Calculations were performed with Microsoft Excel (Microsoft, Redmond, WA, USA) and GraphPad Prism 3.0 (Graph Pad Software, Inc., San Diego, CA, USA). Data are represented as the mean of triplicate values from a single experiment or three independent sets of experiments. Dunnett’s multiple comparison ANOVA test was performed to determine the significant difference between the mean values of samples and the control. Error bars exhibit standard deviation from respective mean values. Significant *p* values were set as * *p* < 0.05,** *p* < 0.01, and *** *p* < 0.001.

## 3. Results

### 3.1. Docking with CYP17A1 and CYP19A1

We performed computational docking and a binding analysis of essential oil compounds against the three-dimensional crystal structures of multiple steroid-metabolizing cytochrome P450 enzymes including CYP11A1, CYP17A1, CYP19A1, and CYP21A2. 

**Figure 5:**
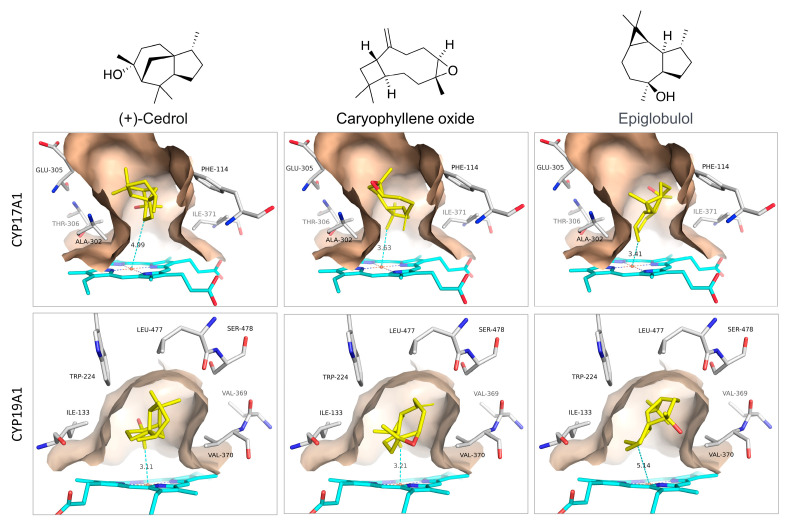
Proposed binding modes of (+)-Cedrol, Caryophyllene oxide, and Epiglobulol to CYP17A1 and CYP19A1.

**Figure 6:**
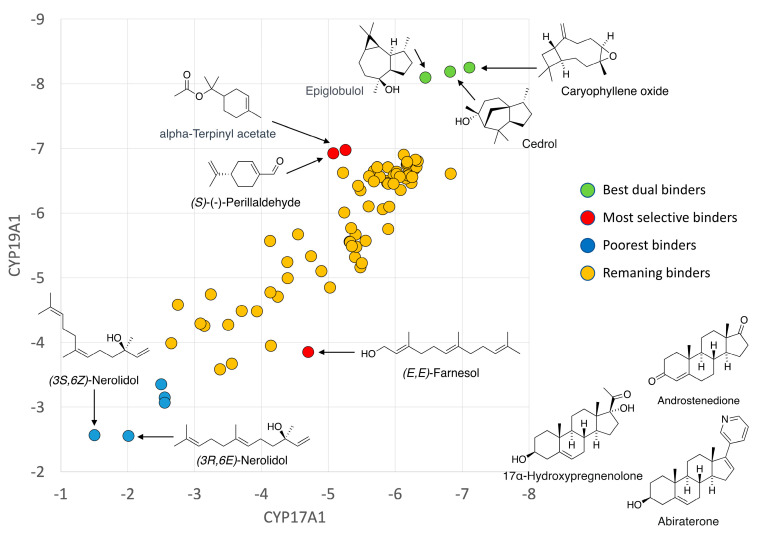
CYP17A1/CYP19A1 selectivity. Plot of binding energies to CYP17A1 and CYP19A1, respectively. Known substrates/inhibitors of CYP17A1 and CYP19A1 were used as controls.

The 25 best binders were clustered into groups based on binding to multiple enzymes and subjected to detailed binding analysis. While we observed no significant selectivity (Figure 4), we identified the generally best potential binders for these CYP enzymes and took a preliminary look at the binding mode of the best poses. The conclusion is that a small number of compounds (Caryophyllene oxide, (+)-Cedrol and Epiglobulol) seem to bind reasonably well to the CYPs primarily by hydrophobic interactions. The compounds bind in the active sites of both CYP17A1 and CYP19A1 without coordinating directly to the Fe atom in the heme group (Figure 5). The binding without heme coordination suggests that while compounds are structurally suitable for binding to CYP active sites, a strong binding and inhibitory effect on CYPs is not expected. Docking studies revealed that (+)-Cedrol was among the best binding compounds to both CYP17A1 and CYP19A1 (Figure 6). The experimental results provided some context to the computational studies. While (+)-Cedrol did not inhibit CYP17A1 activity, it caused 30% inhibition of CYP19A1 activity, and in the best binding pose observed, it was closer to heme in CYP19A1 (3.1 Å) compared to CYP17A1 (4.99 Å) (Figure 5). Dihydro-β-Ionone showed 30% inhibition of both CYP17A1 (17,20 lyase) and CYP19A1 activities. Eucalyptol and (−)-α-pinene showed 20% to 40% inhibition of CYP17A1 17,20 lyase activity. Although the inhibition was weaker than the reference compounds, some of these compounds could be an inspiration for the design of novel inhibitors, since the top scoring poses are rather globular compounds filling the cavity above the heme group in the CYPs. Therefore, it is possible to incorporate some of these structural elements as building blocks in combination with heme-binding nitrogen-containing groups to design novel inhibitors.

### 3.2. Effect on CYP17A1 Activity

**Figure 7:**
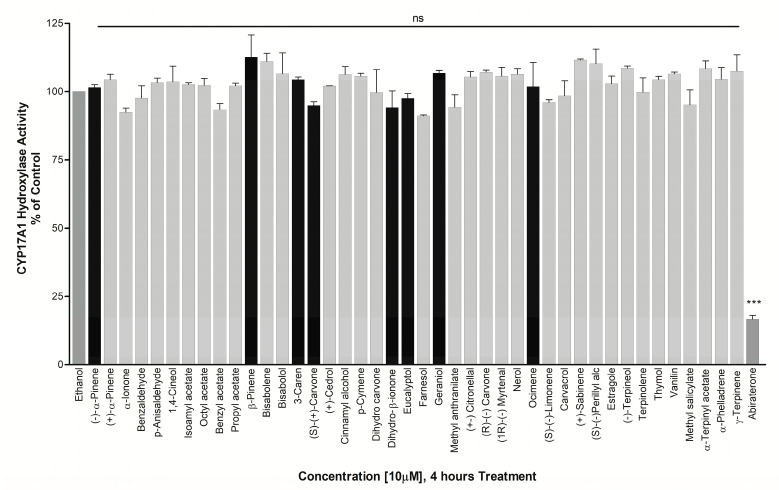
Assay of CYP17A1 17α-hydroxylase activity. Essential oil compounds were tested for effects on CYP17A1 17α-hydroxylase activity using radiolabeled progesterone as substrate and conversion to 17-hydroxy progesterone was monitored using autoradiography of steroids after separation by TLC. The CYP17A1 inhibitor abiraterone was used as a positive control. Effects were calculated as percentage of control. Compounds showing differential activities in different assays are shown as darker bars. ns: not significant, *** *p* < 0.001

Studies in human cell lines [8,13] have shown that lavender oil (LO) and tea tree oil (TTO) act as hormone mimickers for estrogen receptors (ERs) and antagonists for ARs. Moreover, LO and TTO impacted the ER- and AR-mediated regulation of several endogenous genes. Owing to these different mechanisms of action by LO and TTO, we screened several EO components, including the ones found in TTO and LO. In the initial screening of 50 test compounds against CYP17A1 hydroxylase activity, we found no significant effect in NCI H295R cells treated with 10 µM of compounds for 4 h (Figure 7). 

**Figure 8:**
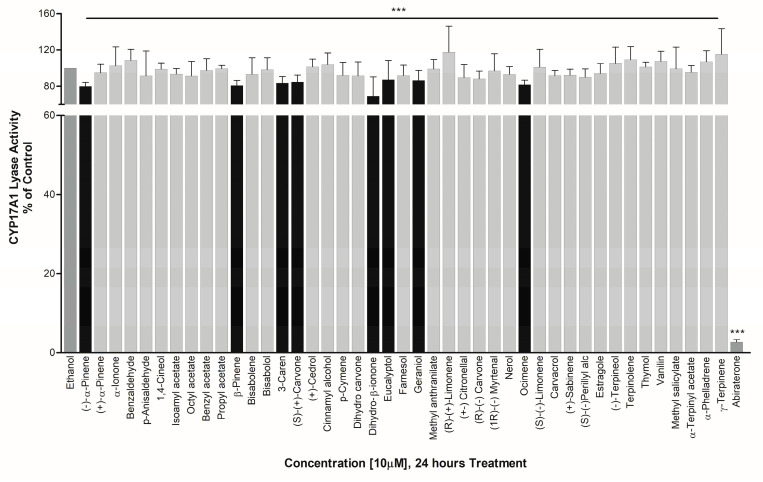
Effect of essential oil compounds of CYP17A1 17,20 lyase activity. Compounds were tested for effects on CYP17A1 17,20 lyase activity using radiolabeled 17OH-Pregnenolone as substrate and conversion to DHEA was monitored using scintillation counting. The CYP17A1 inhibitor abiraterone was used as a positive control. Effects were calculated as percentage of control. Compounds showing differential activities in different assays are shown as darker bars. *** *p* < 0.001

However, essential oils including Eucalyptol, Geraniol, (S)-(+) Carvone, 3-Caren, Ocimene, β-Pinene, (−)-α-Pinene, and Dihydro-β-ionone showed about 13%, 13%, 15%, 16%, 18%, 19%, 20%, and 31% inhibition in CYP17A1 17,20 lyase activity, respectively. The effect of essential oils towards an exclusive inhibition of CYP17A1 17,20 lyase activity makes them good candidates to study further as basic structural leads for designing more potent inhibitors (Figure 8). 

### 3.3. Effect on CYP19A1 Activity

**Figure 9:**
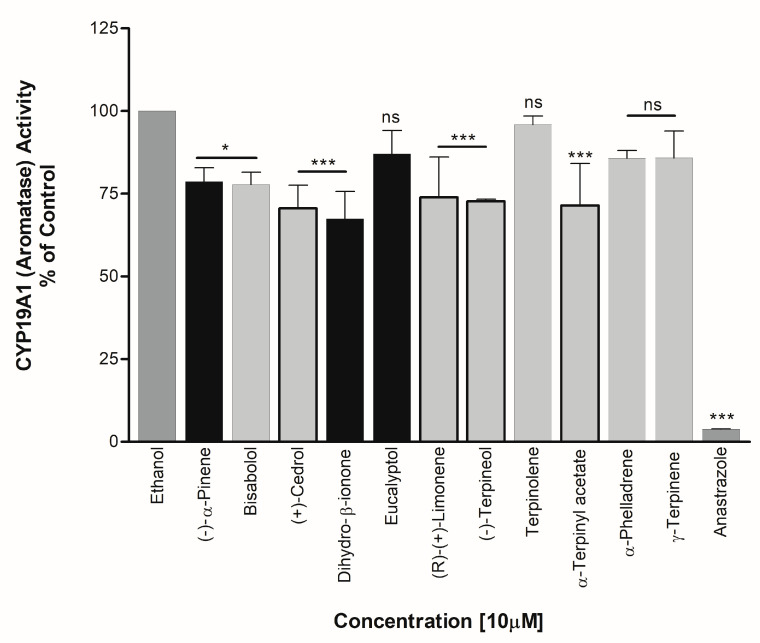
Effects of essential oil components on CYP19A1 activity. Essential oil components were tested against CYP19A1 activity using microsomes prepared from placental JEG-3 cells. Radiolabeled androstenedione was used as substrate and conversion to estrone was monitored by water-release assay. A known CYP19A1 inhibitor, anastrazole, was used as positive control. Compounds showing differential activities in different assays are shown as darker bars. ns, not significant, * *p* < 0.05, *** *p* < 0.001

Essential oils exhibiting significant effects on CYP17A1 activity and those predicted to be estrogenic in nature in some papers in the literature were selected for screening of CYP19A1 activity. Bisabolol, Cedrol, Dihydro-β-ionone, (R)-(+)-Limonene, (−)-Terpineol, and α-Terpinyl acetate showed significant inhibition of aromatase at about 22%, 29%, 33%, 26%, 27%, and 29%, respectively (Figure 9). Dihydro-β-ionone and pinene were found to be effective inhibitors of both CYP17A1 Lyase activity and CYP19A1 activity. 

### 3.4. Effect on PCa Cell Viability

**Figure 10:**
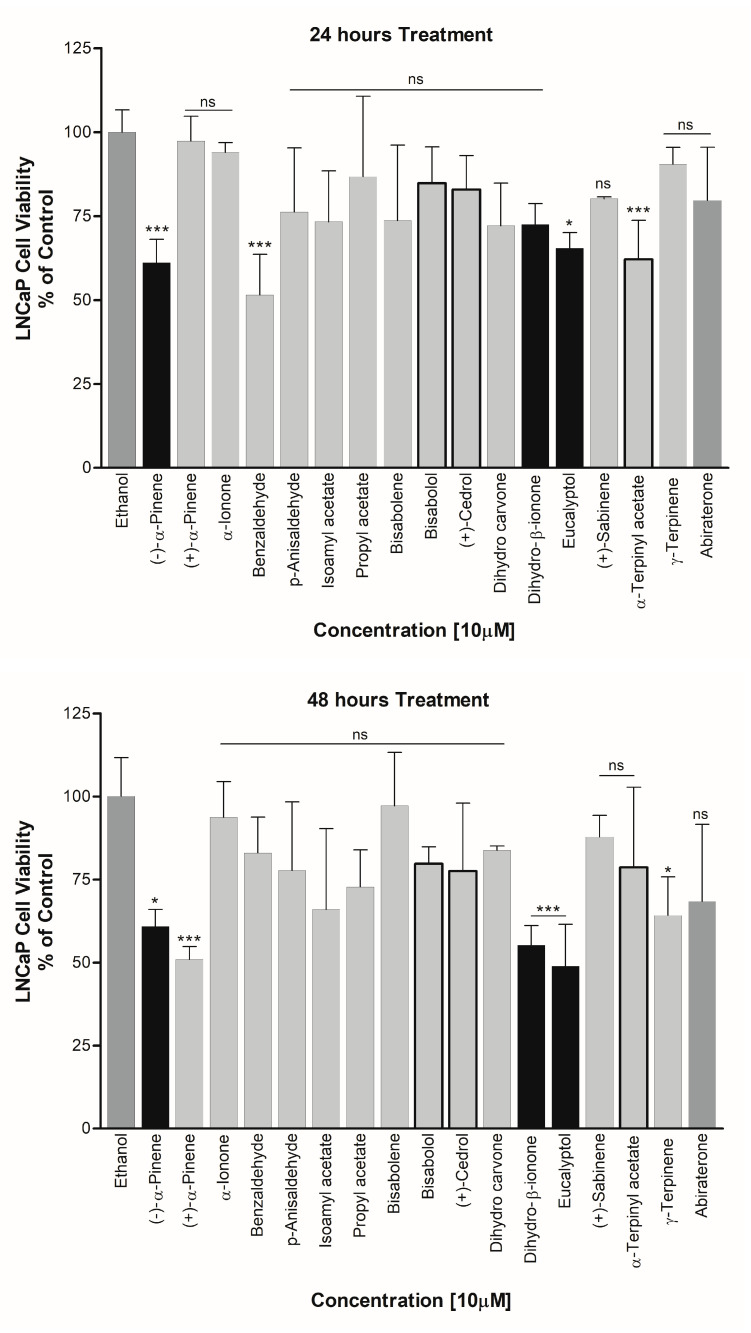
Effect of essential oil components on LNCaP cell proliferation. Essential oil compounds were checked for the effect on proliferation of androgen-dependent PCa cell line on LNCaP cells. Cell viability was determined after 24 h and 48 h treatment with selected compounds that showed inhibitory effects in cytochrome P450 assays. The CYP17A1 inhibitor abiraterone, an anti-prostate cancer drug in clinical use, was used as a positive control. Compounds showing differential activities in different assays are shown as darker bars. ns, not significant, * *p* < 0.05, *** *p* < 0.001

(−)-α-Pinene, Dihydro-β-ionone, and Eucalyptol were also found to cause cell toxicity in the PCa cell line on LNCaP cells. All of these compounds showed an increased potency for cell growth inhibition with increasing treatment durations. Up to 50% reduction in cell viability was observed when the cells were treated with these essential oils for 48 h. Cedrol, which showed significant inhibition of CYP19A1 activity, was also found to reduce the cell viability of LNCaP cells. However, since a direct effect on CYP17A1 activity was not observed by cedrol, a different mechanism may be involved in the toxicity towards LNCaP cells (Figure 10). On the other hand, (−)-α-Pinene, which inhibited 17,20 lyase activity of CYP17A1, showed good inhibition of LNCaP cell proliferation at both 24 and 48 h, suggesting a mechanism at least partially linked to the inhibition of CYP17A1 17,20 lyase activity (Figure 10).

### 3.5. Steroid Analysis by LC-MS/MS

**Figure 11:**
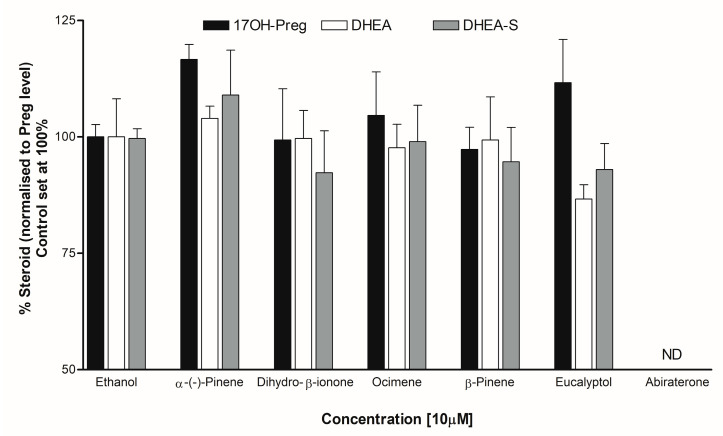
Steroid profiling using LC-MS/MS. Effect of essential oil components was tested for overall effects on steroid profiles of human adrenal NCI-H295R cells. Cells were treated with different compounds and steroid metabolites were analyzed by LC-MS/MS. Results shown are normalized against pregnenolone. The CYP17A1 inhibitor abiraterone was used as a positive control. ND, not detected.

Individual steroid levels were normalized to the amount of Pregnenolone (Preg/P5). Pregnenolone was the starting steroid substrate to profile all the steroids in the biosynthetic pathway. The addition of dihydro-β-ionone to adrenal cells did not alter the levels of 17OH-Pregnenolone (17OH-Preg) or DHEA. However, DHEA-S levels appeared lower (about 8%) compared to the control. The addition of Eucalyptol reduced DHEA levels (about 13%) 1.2-fold (approaching significance at *p* = 0.0574), also with lower DHEA-S levels compared to the control (Figure 11). 

## 4. Discussion

Essential oils are highly concentrated plant extracts that are routinely used in wellness, beauty, and cleaning products. However, since essential oils are not pharmaceutical products, they are not regulated and therefore their safety profiles are a topic of concern due to potential adverse reactions associated with their use. The safety of essential oils may also depend on individual metabolic profiles. In the past, a few studies have been performed about the effect of essential oils on cytochrome P450 enzymes that are involved in drug and xenobiotic metabolism [62]. Spicakova et al. have studied the effect of sesquiterpenes beta-caryophyllene oxide and trans-nerolidol by docking to CYP3A4 and checking the results by functional assays of enzyme activity [63,64]. Beta-caryophyllene oxide, which binds to CYP3A4 close to the heme without coordinating to the Fe atom, showed a week inhibition of CYP3A4 activity. Similarly a week inhibition of CYP2C8, CYP2C9, and CYP2C19 was observed for cedrene, cedrol, and thujopsene, but cedrol showed strong inhibition of CYP3A4 and CYP2B6 [65]. 

Steroidogenesis leads to developmental and reproductive changes, as well as impacting immunological and neurological changes linked to steroid hormones. Here, we have investigated the impact of several essential oil components on steroid production mediated by CYP17A1 and CYP19A1, two key enzymes involved in the regulation of androgen and estrogen in humans. We first performed a computational analysis of the binding of essential oil compounds to two main steroid-metabolizing cytochromes P450, CYP17A1, which is involved in production of androgen precursors, and CYP19A1, which is essential for the biosynthesis of estrogens. A virtual library of eighty-two terpenes, covering the compounds in our chemical library and three control compounds, was subjected to virtual screening. Based on the best binding poses obtained from the initial screening, we selected the 25 best binding compounds for further analysis. We also compared the binding of these terpenes to structurally similar CYP11A1 and CYP21A2 enzymes for comparative analysis of binding affinity or selectivity. With the current analysis, we did not see any specific element of selectivity that results in better binding to a particular cytochrome P450 but found several terpenes that bind close to the heme group of cytochrome P450 enzymes in our analysis, without a direct interaction with heme iron that is observed for strong binding inhibitors. Combined with the inhibition of CYP19A1 observed for bisabolol, cedrol, dihydro-β-ionone, (R)-(+)-limonene, (−)-terpineol, and α-terpinyl acetate, and the inhibitory effects of Eucalyptol, geraniol, (S)-(+) carvone, 3-caren, ocimene, β-pinene, (−)-α-pinene, and dihydro-β-ionone on CYP17A1, it is possible that structural elements of these terpenes could be combined with strong heme iron coordinating groups to produce inhibitors that bind selectively to these CYPs. The inhibition of individual CYPs may depend on competition with substrates and although the terpenes used in our study did not result in strong inhibition, the selectivity for the CYP17A1 17,20 lyase reaction shown by some of the terpenes warrants further exploration.

In addition, we saw a significant anti-proliferative effect of (−)-α-Pinene, dihydro-β-ionone, and Eucalyptol on LNCaP prostate cancer cells, which suggests that multiple different mechanisms may be involved, and further experiments on cell cycle regulation, nuclear receptor antagonism, and gene expression analysis may reveal further details regarding the mechanism of the anti-cancer properties of these essential oils. 

**Figure 12:**
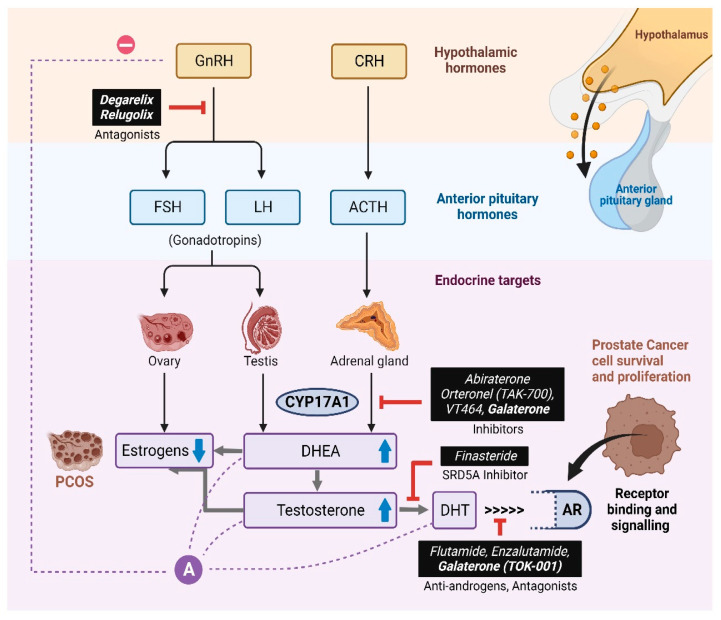
Androgen production and its role in PCOS and PCa and different approaches to target androgen production. Several different approaches are being currently being pursued to target androgen production for the treatment of hyperandrogenic disorders. These include GnRH antagonists, CYP17A1 inhibitors, 5α-reductase inhibitors, and androgen receptor-targeting chemicals. Created by https://www.Biorender.com.

Currently, the classical methods employed in the treatment of PCa include androgen deprivation therapy using CYP17A1 inhibitors and/or blocking AR binding to its ligand using AR antagonists [66,67] (Figure 12). CYP17A1-targeted drugs/drug candidates have been developed over the years for the treatment of PCa, as well as Castration-Resistant PCa (CRPC) [68].CYP17A1 has emerged as an attractive target for the design of inhibitors to use as drugs against PCa [69]. From a non-selective cytochrome P450 inhibitor, ketoconazole, and first-generation CYP17A1-targeted drugs such as abiraterone and orteronel (TAK700), to the most recent compounds with better selectivity towards 17,20 lyase activity like galeterone (TOK-001) and VT464, there is a continuous ongoing search for more efficient and potent inhibitors to overcome the challenges resulting from the adverse side-effects of existing compounds [70,71,72,73,74,75,76,77,78]. For instance, in addition to CYP17A1, abiraterone also targets cytochrome P450 21-hydroxylase (CYP21A2) activity, which is essential for aldosterone and cortisol production [79,80]. As a result, suboptimal levels of cortisol due to inhibition of CYP21A1 lead to the requirement of glucocorticoid co-therapy in these patients [81]. Current strategies for the development of drugs focus on designing inhibitors with the ability to modulate elevated levels of circulating androgens [82,83], as well as steroids derived from alternative pathways in the case of androgen-independent PCa without disturbing the cortisol metabolism [84,85]. Therefore, in the search for novel inhibitors of CYP17A1 with improved target specificity and reduced off-target effects, essential oils could be utilized as building blocks for designing novel drugs against PCa and PCOS.

## Data Availability

Data are available in the manuscript text or in the supplementary materials.

## Supplementary Materials

The following supporting information can be downloaded at: https://www.mdpi.com/article/10.3390/biom14020203/s1, File S1: Docking analysis of terpenes.
